# AphaMax^®^, an Aphanizomenon Flos-Aquae Aqueous Extract, Exerts Intestinal Protective Effects in Experimental Colitis in Rats

**DOI:** 10.3390/nu12123635

**Published:** 2020-11-26

**Authors:** Maria Grazia Zizzo, Gaetano Caldara, Annalisa Bellanca, Domenico Nuzzo, Marta Di Carlo, Stefano Scoglio, Rosa Serio

**Affiliations:** 1 Department of Biological, Chemical and Pharmaceutical Sciences and Technologies (STEBICEF), University of Palermo, Viale delle Scienze, 90128 Palermo, Italy; gaetanofelice.caldara@unipa.it (G.C.); rosa.serio@unipa.it (R.S.); 2 ATeN (Advanced Technologies Network) Center, Viale delle Scienze, University of Palermo, 90128 Palermo, Italy; annalisa.bellanca@unipa.it; 3 Institute of Biomedicine and Molecular Immunology “Alberto Monroy” (IBIM), Consiglio Nazionale delle Ricerche (CNR), 90146 Palermo, Italy; domenico.nuzzo@irib.cnr.it (D.N.); marta.di.carlo@irib.cnr.it (M.D.C.); 4 Nutritherapy Research Center, 61029 Urbino, Italy; stefanoscoglio@me.com

**Keywords:** inflammation, Aphanizomenon flos-aquae, blue-green algae, inflammatory bowel disease

## Abstract

Background: Aphanizomenon flos-aquae (AFA) is a unicellular cyanobacterium considered to be a “superfood” for its complete nutritional profile and beneficial properties. We investigated possible beneficial effects of an AFA extract, commercialized as AphaMax^®^, containing concentrated amount of phycocyanins and phytochrome, in 2,4 dinitrobenzensulfonic acid(DNBS)-induced colitis in rats. Methods: Effects of preventive oral treatment of AphaMax^®^ (20, 50 or 100 mg/kg/day) in colitic rats were assessed and then macroscopic and microscopic analyses were performed to evaluate the inflammation degree. Myeloperoxidase (MPO) activity and NF-κB, pro-inflammatory citockines, cycloxygenase-2 (COX-2), and inducible NOS (iNOS) levels of expression were determined, as Reactive Oxygen Species (ROS) and nitrite levels. Results: AphaMax^®^ treatment attenuated the severity of colitis ameliorating clinical signs. AphaMax^®^ reduced the histological colonic damage and decreased MPO activity, NF-κB activation, as well as iNOS and COX-2 expression. AphaMax^®^ treatment improved the altered immune response associated with colonic inflammation reducing IL-1β, IL-6 expression. Lastly, AphaMax^®^ reduced oxidative stress, decreasing ROS and nitrite levels. Conclusions: Preventive treatment with AphaMax^®^ attenuates the severity of the inflammation in DNBS colitis rats involving decrease of the NF-kB activation, reduction of iNOS and COX-2 expression, and inhibition of oxidative stress. Due its anti-inflammatory and antioxidant proprieties AphaMax^®^ could be a good candidate as a complementary drug in inflammatory bowel disease (IBD) treatment.

## 1. Introduction

Inflammatory bowel diseases (IBDs), are chronic and non-resolving intestinal inflammatory diseases, which include two clinical entities: Crohn’s disease (CD) and ulcerative colitis (UC). Their etiology is far from being fully understood, although it is widely recognized that genetic, environmental, microbial, and immune factors are functionally integrated in the IBD pathogenesis [1]. A pivotal role seems to play a worsening and inappropriate mucosal immune response. In particular, NF-κB activation, production of pro-inflammatory cytokines such as IL-6 and IL-1β [2,3,4], myeloperoxidase (MPO), expression of cycloxygenase-2 (COX-2), and inducible nitric oxide synthase (iNOS) collectively enhance inflammatory response in the colon and lead to a concomitant reduction in antioxidant levels. These molecular events could culminate in a serious damage of epithelial cell and disruption of the mucosal barrier [5,6].

Conventional IBD therapies have various side effects, and many patients do not respond to these treatments [7]. Thus, to identify effective new therapeutic options for improving colitis signs with fewer or no side effects, has becoming essential.

In recent years, attention was increased on complementary medicine approaches based on natural ingredients.

Various bioactive compounds, such as phycocyanins, carotenoids, γ-linolenic acid, fibers, and plant sterols, have been reported to clinically improve IBD-related symptoms [8]. However, the efficacy and mechanism of action of these products require further studies in vitro and in vivo models. The cyanobacteria blue-green algae (BGA), are photosynthetic prokaryotes present in aquatic ecosystems, rich in these bioactive molecules [9,10]. Recent studies highlight that their consumption is associated with ameliorative effects on the components of human metabolic syndrome [11], to the decrease of inflammation markers in hypertensive patients [10], as well as in obese patients. Moreover, immunomodulatory effects of BGA supplementation has been shown in healthy volunteers [12]. Aphanizomenon flos-aquae (AFA) also known as “Klamath algae”, is as cyanobacterial dominant species growing in the Upper Klamath Lake (Oregon, USA) [13].

AFA, consumed as a “superfood”, is a rich source of mycosporine-like amino acids (MAAs) and phycocyanins (PCs), likely responsible for its various health benefits. AphaMax^®^ is a unique patented extract from AFA, manufactured by Nutrigea, containing a concentrated amount of AFA-phycocyanins and AFA-phytochrome, the Klamath microalgae components showing the greatest antioxidant, anti-inflammatory, anticancer, and cardiovascular properties. These molecules, which are unique to Klamath microalgae, increased the beneficial proprieties compared to other blue-green algae. Indeed, AFA-PCs due to their peculiar structure [14] have up to 200 times higher antioxidant power compared to other PCs [15]. AFA-PCs have neuroprotective effects and ameliorate psychological stress and on menopausal well-being [16] In addition, AphaMax^®^ is rich in some smaller molecules, endowed with similar antioxidant properties, as many as the AFA-mycosporines (MAAs, or mycosporine-like amino acids) [17], 15 carotenoids, including zeaxanthin, lutein, canthaxanthin, a wide spectrum of polyphenols as caffeic acid, and an high content of chlorophyll.

Given the well-demonstrated anti-inflammatory and antioxidant effects of the majority of the molecules present in Klamath algae, as phycocyanins that selectively inhibit key enzymes in inflammatory disease as COX-2, and iNOS, and the high concentration of these components in AphaMax^®^, the aim of our study was to investigate the possible protective potential of this extract in an animal model of IBD, the 2,4-dinitrofluorobenzenesulfonic acid (DNBS) -induced rat colitis. DNBS rat colitis shares many of the pathological features of human Crohn’s disease and is considered useful for studying the etiopathogenesis of IBD as well as for providing an inexpensive model useful for investigating new potential treatments [18].

## 2. Materials and Methods

### 2.1. Animals

Thirty healthy adult Wistar male rats (8–9 weeks old, 250–300 g) were purchased from ENVIGO Srl (San Pietro al Natisone UD, Italy) and housed randomly in temperature-controlled rooms on a 12h light cycle at 22–24 °C and 50–60% humidity. Animals had free access to standard pellet chow and water ad libitum throughout the experimental protocol. They were acclimatized for one week prior to experimentation. The experimental protocol, followed throughout the study, was conducted in the conformity of the Italian D.Lgs 26/2014”, following the criteria outlined by the European Community Council Directive 2010/63/UE, recognized and adopted by the Italian Government, and approved by the Ethical Committee for Animal Experimentation of the University of Palermo and by the Italian Ministry of Health (Authorization n 921/2018–released Rome, Italy).

### 2.2. Induction of DNBS Colitis and Treatment Protocol

AphaMax^®^ extract (Nutrigea Research s.r.l., Borgo Maggiore, Republic of San Marino) was dissolved in 0.5 mL water, and then sonicated (twice for 60 s) immediately prior to the gavage. 20, 50 or 100 mg/kg AphaMax^®^ solution was administered by oral gavage once a day for 14 days starting 7 days before the induction of colitis (day-7). Rats were randomly assigned into six groups (*n* = 5 animals/each): (1) control group (sham group); (2) group with colitis; (3) sham group + AphaMax^®^ (100 mg/Kg/day); (4) group with colitis + AphaMax^®^ (20 mg/Kg/day); (5) group with colitis + AphaMax^®^ (50 mg/Kg/day); (6) group with colitis + AphaMax^®^ (100 mg/Kg/day). To induce the colitis intracolonic (i.c.) instillation of 2, 4-dinitrobenzensulfonic acid (DNBS; Sigma-Aldrich Inc., St Louis, MO, USA), was performed as already described [19,20,21]. Briefly, rats were fasted overnight and then, under light anesthesia with 1% isoflurane (Merial Italia Spa, Assago, MI, Italy), DNBS (15 mg) dissolved in a solution of 50% ethanol, was deposited in the colon through an 8 cm plastic catheter (PE90).

To avoid reflux actions, the rats were kept for 5 min in a Trendelenburg position, and then allowed to recover with food and water supplied. The control group (sham group) received i.c instillation of vehicle alone (50% ethanol).

None of the rats died during the study or was excluded from the study for any reason. Assessment of colitis-induced damage was performed minimizing the suffering of animals, in a blinded fashion as previously described [18,19,20,21].

### 2.3. Evaluation of Colitis

#### 2.3.1. Monitoring of Change in Body Weight, Consistency of Stools and Rectal Bleeding

The animals were monitored daily during the experimental period and scored for body weight loss percentage (initial body = 100%), stool consistency and the presence of rectal blending. All these parameters were ranged and calculated as described by Cooper et al. [22].

#### 2.3.2. Macroscopic Scores

Seven days after colitis induction, rats were sacrificed, and laparotomy was performed and the appearance of colon was then examined. Distal colon was rapidly removed, opened longitudinally and gently washed with saline. For each specimen wet weight (mg)weight/length (cm) ratio was calculated as indicator of colonic edema. For macroscopic damage, each animal was scored by Appleyard and Wallace classification system score [23], The sum of scores for the ulceration (0 = No mucosal damage, 1 = Localized hyperemia but no ulcers, 2 = Ulcers without hyperemia/bowel wall thickening, 3 = Ulcers with hyperemia/bowel wall thickening at 1 site, 4 =Two or more sites of ulceration or inflammation, 5 Area of damage (necrosis) extended >1 cm along length of colon, 6–10 Area of damage extended >2 cm along length of colon; increasing the score was by 1 for each additional cm involved) for the adhesions (0 = No adhesions, 1 = Minor adhesions (no difficult separation of the colon from the other tissue), 2 = Major adhesions) and for the evaluation of thickness (Wall thickness(x) of bowel calculated in mm) were calculated.

#### 2.3.3. Analysis of Microscopic Inflammatory Damage

The colon tissues of rats were fixed in 4% formaldehyde (Sigma–Aldrich, Inc., St. Louis, MO, USA), embedded in paraffin for histological studies and sectioned (5 μm thick). The slices were stained with haematoxylin and eosin (Bio-Optica Milano SpA, Milano, Italy). Histological sections were examined in a blinded fashion, and photos of sections were taken by light microscope (Olympus BX50, Olympus Optical Co., Tokio, Japan). The microscopic damage following score was calculated the Hunter et al. [24] classification method: by adding the histological finding (0 = normal, 1 = minimal, 2 = mild, 3 = severe), the degree of inflammatory infiltration (0 = normal, 1 = minimal, 2 = mild, 3 = severe), the layers infiltrated (0 = normal, 1 = minimal, 2 = mild, 3 = severe), the mucosal damage (0 = normal,1 = minimal, 2 = mild, 3 = severe), and the edema in the mucosa (0 = absent, 1 = present).

### 2.4. Assay of MPO Activity

The activity of myeloperoxidase (MPO) is an important marker for inflammatory damage. The MPO activity was estimated spectrophotometrically using hydrogen peroxide and o-dianisidine as substrate for MPO enzyme, as previously described [20,21] by following a method of Moreels et al. [25] The absorbance value was read at 460 nm (Beckman-Coulter Inc, Brea, CA, USA). MPO activity was expressed as units per gram tissue (U gram tissue^−1^), taken that 1 unit of enzyme reduces 1 µmole hydrogen peroxide (H_2_O_2_) per minute.

### 2.5. ELISA Assay for Pro-Inflammatory Cytokines

Commercial ELISA Kits (Cloud-Clone Corp, Wuhan, Hubei, China) was used for the evaluation of colonic amounts of interleukin-1β and interleukin-6 according to the manufacturer’s instructions, as previously described [20,21].

### 2.6. NFκ-B, iNOS and COX-2 mRNA Analysis by Real-Time PCR

Total RNA was extracted from colon tissues (10 mg) using RNAeasy Mini kit (Qiagen, Valencia, CA, USA). 2 ng of total RNA were reverse transcribed into cDNA using RT FirstStrand kit (Qiagen, Valencia, CA, USA). The amplification of synthesized cDNAs was performed using SYBR Premix Ex Taq II (TaKaRa, Bio Inc., Foster City, CA, USA) and StepOne Real-Time instrument (Applied Biosystems, Foster City, CA, USA). Gene expression of inducible NOS (iNOS), COX-2 and beta actin, a housekeeping gene that is not subject to regulation, was performed in triplicate, using specific primers and amplification conditions. The oligonucleotide primer sequences were reported in Table 1.

The program for PCR was 1 cycle of 95 °C (10 min), followed by 45 cycles of amplification. Consisting of denaturation at 95 °C (15 s), annealing at 60 °C (30 s), and extension for at 72 °C (30 s). For terminal elongation period, the samples were incubated at 72 °C (additional 10 min) at the end of the final cycle. The expression level was calculated from the PCR cycle number (CT) where the increased fluorescence curve passes across a threshold value. The relative gene expression of the target genes was calculated using 2^−ΔΔCt^ approximation method algorithm.

### 2.7. Analysis of Reactive Oxygen Species (ROS) Generation

The conversion of non-fluorescent DCFH-DA to 2′, 7′ dichlorofluorescein (DCF) was evaluated, as previously described [20,21], to monitor the amount of hydrogen peroxidase in the sample. Sample was analyzed by fluorimeter (Microplate reader WallacVictor 2-1420 Multilabel Counter; PerkinElmer, Inc., Waltham, MA, USA) at an excitation wavelength of 485 nm and an emission wavelength of 530 nm.

### 2.8. Nitric Oxide (NO) Concentration Assay

Nitric oxide concentration was measured using the Griess reaction [30]. Briefly after homogenization of rat colon tissue (10 mg) with 1 mL PBS, pH 7.2 and centrifugation (14,000 rpm, for 30 min, 4 °C) the supernatant was incubated with equal volume of Griess reagent (room temperature, 15 min in the dark) and the absorbance was measured at 550 nm with a spectrophotometric Microplate reader (WallacVictor 2 Multilabel Counter, Perkin Elmer, Apeldoorn, The Netherlands) NO concentration was evaluated by using a standard curve.

### 2.9. Data Analysis and Statistical Tests

Results are shown as the mean ± SEM: ‘*n*’ indicates the number of animals. Statistical analysis was performed using GraphPad Prism 6.0 software, and sets were assessed by one-way ANOVA followed by Tukey’s multiple comparison test. *p* value < 0.05 means statistically significant. To compute the sample size G*Power version 3.1.2 [31], was used, given power (1 − β) = 0.8.

## 3. Results

DNBS challenge in rats induced a substantial decrease in the body weight starting by day 2 following the enema, in contrast to the sham group which showed body weight gain. During the same period DNBS group experienced diarrhea and also rectal bleeding was observed. AphaMax^®^ (20–100 mg/Kg for 14 day) dose-dependently prevented the drop in the animal weight and improved the diarrheal status and bleeding in DNBS treated rats (Table 2). 

### 3.1. AphaMax^®^ Effects on Macroscopic Changes in Colon of Colitis Animals

**Figure 1:**
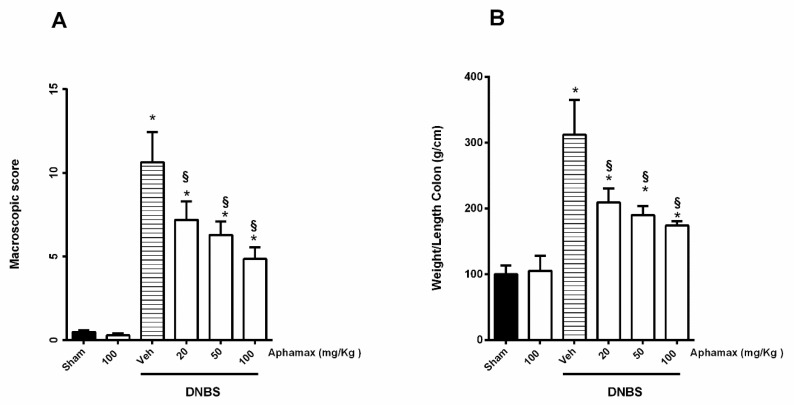
Effects of Aphamax^®^ on DNBS-induced macroscopic colonic damage. (**A**) Macroscopic damage score based on Appleyard and Wallace method and (**B**) Colon weight/Length ratio in sham rats or in Aphamax^®^-treated rats with or without colitis induction. Data are means ± S.E.M. *n* = 5 animals /group. * *p* < 0.05 *versus* Sham animals; ^§^ *p* < 0.05 *versus* 2,4 dinitrobenzensulfonic acid (DNBS) animals.

Macroscopic evaluation demonstrated that in the sham group, the distal colon showed no epithelial damage differently from colitis group in which intense mucosal damage, increased wall thickness, hyperemia, ulceration, edema and necrosis were observed associated with high macroscopic score (Figure 1A). DNBS rats revealed also colon shortening accompanied by an increased in the ratio colon weight/length, a marker of tissue inflammation, (13.27 ± 0.46 cm vs. 9.75 ± 0.41 cm colon length in Sham group and in DNBS group respectively *p* < 0.05; 1330.01 ± 120.3 mg vs. 3167.10 ± 443.21 mg colon weight in Sham group and in DNBS group respectively *p* < 0.05) (Figure 1B). AphaMax^®^ (20, 50, or 100 mg/kg) treatment decreased the DNBS-induced macroscopic changes by improving the inflammation symptoms in colon tissues such as mucosal injury, size of ulcer area and also a reduction of the colon weight/length ratio, (11.25 ± 0.45; 12.16 ± 0.65 and 12.91 ± 0.47 cm in colon length in DNBS + AphaMax^®^ 20, 50, or 100 mg/kg respectively ; 2447.03 ± 147.51 mg, 2141.66 ± 80.01 mg, 2095.01 ± 122.03 mg colon weight in in DNBS + AphaMax^®^ 20, 50, or 100 mg/kg respectively, Figure 1B). Diffuse adhesions of the colon with other organs, typically observed during the acute phase of colitis, were unremarkable and only sporadically observed in AphaMax^®^-treated animals at all doses tested.

AphaMax^®^ *per se*, at the highest dose used, 100 mg/Kg, had no effect on sham animals (Figure 1A,B).

### 3.2. AphaMax^®^ Effects on Histopathological Changes in Inflamed Colon Tissue

**Figure 2:**
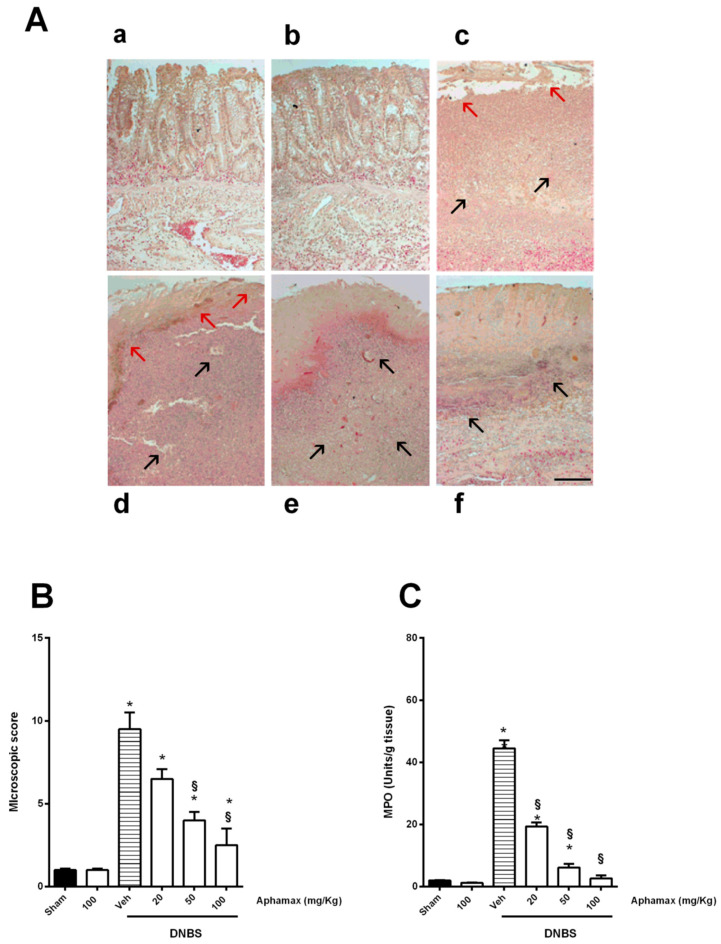
Effects of Aphamax^®^ on DNBS-induced histological damage. (**A**) Photomicrographs of the colon stained with H&E from (**a**) Sham animals, (**b**) Sham rats treated with Aphamax^®^ (100 mg/kg), (**c**) DNBS animals showing colonic damage and a diffuse infiltration of inflammatory cells in mucosa and submucosa or (**d**) DNBS rats treated with Aphamax^®^ (20 mg/kg), (**e**) or with Aphamax^®^ (50 mg/kg), showing progressive reduction of submucosal infiltration of inflammatory cells in submucosal layer and (**f**) DNBS rats treated with Aphamax^®^ (100 mg/kg) showing few inflammatory cells close to the mucosal layer. (Scale bar = 100 μm, magnification 20×, red arrows = colonic damage, black arrows = inflammatory infiltrate)) (**B**) Microscopic damage scored with Hunter method and (**C**) activity of colonic myeloperoxidase in sham rats or Aphamax^®^ treated rats with or without colitis induction. Data are means ± S.E.M. *n* = 5 animals for each group. * *p* < 0.05 *versus* Sham group; ^§^ *p* < 0.05 *versus* 2,4 dinitrobenzensulfonic acid (DNBS) group.

Histological analyses showed in the sham group a colon tissue with normal mucosa, submucosa, crypts, muscularis, lamina propria, and serosa, without inflammatory cell infiltration and necrosis. A score of the histological changes in colitis-induced rats was calculated, as previously described [18,19,20,21] (Table 2). In the DNBS group, the colon showed serious histological changes with high scores of microscopic damages, characterized by large ulcers with necrosis, transmural inflammation, disrupted crypts and massive infiltration into lamina propria and submucosa of inflammatory cells, consisting of lymphocytes, macrophages, and neutrophils. A net distinction of the colon layers from one another was not allowed AphaMax^®^ (20–100 mg/Kg) treatment induced a progressive reduction of inflammatory cell infiltration and decreased the DNBS-mediated colonic damages leading to an improvement in the microscopic damage score; however no one of the concentrations tested completely resolved the histological changes (*p* < 0.05 compared to sham group) (Figure 2A,B).

### 3.3. AphaMax^®^ Effects on MPO and Cytokine Levels

**Figure 3:**
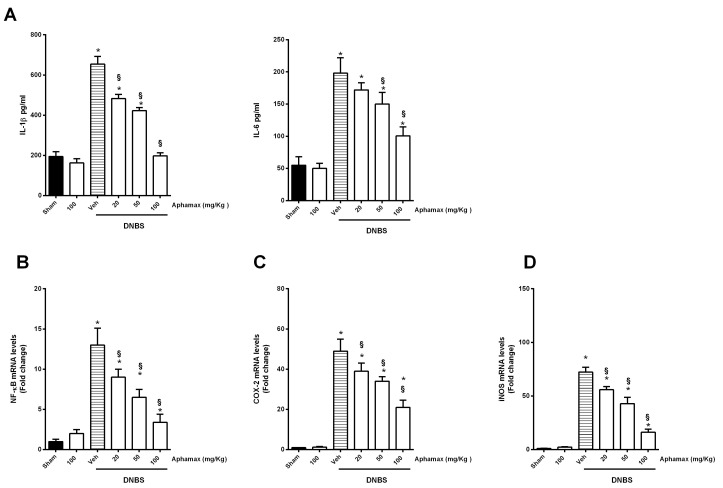
Effects of Aphamax^®^ on pro-inflammatory mediator levels (**A**) Levels of IL-1β, IL-6 levels; (**B**) NF-kB, (**C**) COX-2 and (**D**) iNOS mRNA expression. Data are means ± S.E.M. *n* = 5 animals for each group. * *p* < 0.05 *versus* Sham group; ^§^ *p* < 0.05 *versus* 2,4 dinitrobenzensulfonic acid (DNBS) group.

To explore the possible action mechanism of AphaMax^®^ extract, we examined, at first, the myeloperoxidase levels, index of a massive infiltration of neutrophil into the inflamed tissue. In DNBS animals, the MPO activity was significantly increased compared to the sham group. MPO activity was reduced by AphaMax^®^ treatment in a dose-dependent manner, confirming the histological observation showing a lower infiltration of leukocytes in the colonic tissue (Figure 3C). Moreover, we evaluated the production of proinflammatory cytokines, highly involved in IBD, such as IL-1β and IL-6. In accordance with the immune cell infiltration detected in the histological examination of the DNBS colon, increased levels of IL-1β, IL-6 in comparison to sham animals were observed. Moreover, the reduction in the histological score was correlated with a significant reduction of the levels of IL-1β and IL-6 in AphaMax^®^-treated animals, compared with DNBS group, being however still significantly higher compared to sham group (*p* < 0.05) (Figure 3A).

### 3.4. AphaMax^®^ Effect on NF-κB p65, COX-2 and iNOS mRNA Expression

We assessed the expression of the activated NF-κB p65 subunit, which plays a crucial proinflammatory role during the pathogenesis of IBD. The colon from the DNBS group showed an extensive increase in the mRNA expression of NF-κB p65. NF-κB p65 subunit mRNA expression was significant decreased in AphaMax^®^-treated animals suggesting that a down-regulation of these proinflammatory factors could be implicated in AphaMax^®^ beneficial protective effects (Figure 3B).

As already mentioned, the beneficial effects of blue-green algae could be partly ascribed to the ability of their components to selectively inhibit key enzymes in inflammatory disease as COX-2 and iNOS [32]. Thus, we analyzed mRNA expression of both enzymes in the colonic tissues from the different animal groups. DNBS group showed an increased expression of both enzyme compared to control group, which was reduced after AphaMax^®^ treatment in dose-dependent manner, (*p* < 0.05 compared to DNBS and to sham group) (Figure 3C,D).

### 3.5. AphaMax^®^ Effect on Nitrite and ROS Production

Lastly, to investigate possible antioxidant proprieties of AphaMax^®^ we evaluated if the treatment could affect the tissue NO and ROS generation.

**Figure 4:**
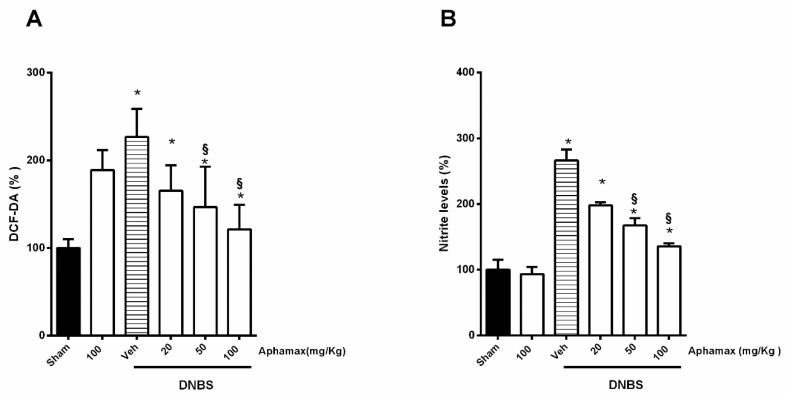
Effects of Aphamax^®^ on ROS and nitrite levels. (**A**) ROS levels measured using DCFH-DA, a peroxide/redox-sensitive fluorescent method. (**B**) nitrite levels detected using Griess assay. Data are means ± S.E.M. *n* = 5 for each group. * *p* < 0.05 *versus* Sham group; ^§^ *p* < 0.05 *versus* 2,4 dinitrobenzensulfonic acid (DNBS) group.

DNBS induced a significant increase of the levels of nitrites and ROS in the colon of DNBS group compared with sham group (*p* < 0.05). AphaMax^®^ at the different doses tested induced a moderate (*p* < 0.05 compared to sham groups) but significant down-regulation in the production of both nitrites and ROS in colitis rats (*p* < 0.05 compared to DNBS group) (Figure 4A,B).

## 4. Discussion

The use of microalgae recently attracted considerable attention worldwide due to their considerable application potential in the biopharmaceutical and nutraceutical industries. They are renewable, sustainable, and inexpensive sources of bioactive molecules and food with surprising pharmacological and biological qualities. In particular, blue-green algae were demonstrated to have remarkable antioxidant and anti-inflammatory properties due to the presence of many bioactive substances [9,10,11,12]. Among the blue-green algae extracts, AphaMax^®^ contains high concentrated amount of AFA-phycocyanins and AFA-phytochrome, which are the components with the greatest antioxidant, anti-inflammatory, anticancer, and cardiovascular properties.

The aim of this study was to test the effects of AphaMax^®^, a commercial extract of the blue-green Klamath microalgae (AFA), on colonic inflammatory condition induced in an experimental model of colitis, the DNBS rats.

The rationale for this comes by the awareness that the current IBD therapies have significant side effects and modest results for long-term management and that recently, much evidence supports the key role of diet and nutritional factors in IBD. Our results indicate the AphaMax^®^ supplementation is able to reduce colon injury induced by DNBS in rats, mainly due to its antiinflammatory and anti-oxidant effects.

As previously reported [18,19,20,21], intracolonic administration of DNBS causes an acute colitis in rats, characterized by marked reduction in body weight, diarrhoea with occasional blood in the stool, colonic shortening, an augmented colonic wall thickness, with a significant increase in the colonic weight/length ratio, reliable indicator of tissue oedema, and an extensive transmural, granulomatous inflammation of the distal colon.

Fourteen days of AphaMax^®^ treatment leaded to a reduction of the aforementioned parameters as the deterioration of body weight, diarrhoea, and the increased colon weight/length ratio. Also histological analysis of the colon confirmed the macroscopic observations, suggesting that AphaMax^®^ presents an antiulcerogenic effect, as confirmed by preservation of the colon architecture and by the attenuation of mucosal disruption, as well as of histopathological damage and oedema.

Few studies have been conducted on the possible protective effects of BGA against experimental colitis, but this is the first study about the effect of Klamath algae in such conditions. Oral administration of cyanobacterium Spirulina Platensis was reported to have protective effects in other models of experimental colitis showing both antioxidant (reduction in oxidative stress and augmented endogenous antioxidant mechanisms) and anti-inflammatory (reduction in inflammatory cytokine levels and neutrophil infiltration) effects [33]. Moreover, in ulcerative colitis patients, diet supplementation with the green algae Chlorella pyrenoidosa accelerated wound healing [34].

Furthermore, we can speculate about the mechanisms underlying the intestinal beneficial effects of the AFA extract. Among the possible mechanisms we can suppose a relation with the well-known positive properties, ascribed to the majority of the blue-green algae extracts, due to their content in compounds, as phycocyanins.

In particular, Klamath algae contains the pigment C-phycocyanin (C-PHY) bound to a structural component phycoerythrocyanin (PEC). PEC is a photosynthetic component identified only in a limited number of cyanobacterial species, likely responsible for the higher antioxidant and anti-inflammatory effects of Klamath algae compared to other cyanobacters [14,15]. C-phycocyanins can act as radical scavenger in oxidative stress-induced diseases and they have strong antioxidant and anti-inflammatory proprieties as demonstrated by various in vitro and in in vivo evidences [35,36].

Moreover, in our experimental model of colitis, AphaMax^®^ treatment induced an attenuation of the neutrophil infiltration, as shown by both histological observations and biochemical data showing a significant reduction in colonic myeloperoxidase activity, confirming the ability of this extract to modulate the altered immune response, likely ascribable to the C-phycocyanins action. This observation is in agreement with Gonzalez et al. [37] where C-phycocyanins extracted from the Blue-green algae Arthospira maxima, have been reported to reduce significantly myeloperoxidase activity, inflammatory cell infiltration and colonic damage in acetic acid-induced colitis in rats. We are aware that the model of colitis generation in Gonzales et al. [37] is different from our model; however, it is possible to observe that in our study the highest doses of AphaMax^®^ (25% AFA-PC concentration) was more effective in the reduction of myeloperoxidase activity of Spirulina (80% PC concentration) indicating the major anti-inflammatory and protective power of AFA-phycocyanins.

Although in our study, AphaMax^®^, at all doses tested, attenuated the colitis signs in the colonic tissue, we can suggest that the higher dose tested, 100 mg/Kg, containing a concentration of AFA-phycocyanins and AFA-phytochrome, carotenoids and polyphenols able to significantly improve all the analyzed scores, could be considered the best to attenuate the severity of colitis.

Moreover, the observation that AphaMax^®^ extract, even at the highest dose tested, did not affect sham rats, might indicate that AphaMax^®^ extract exerts its anti-inflammatory and antioxidant effects, only in the course of inflammation.

Interestingly, AphaMax^®^ treatment is sufficient to attenuate activation of NF-κB p65. NF-κB, is a redox-sensitive transcription factor, key regulator of inflammation, innate immunity, and tissue integrity. NF-κB phosphorylation and its nuclear translocation correlate with the severity of intestinal inflammation [38,39] due to the regulation of gene expression of molecules playing a pivotal role in inflammation as molecules of adhesion, chemokines, and cytokines [40]. We can suppose that the attenuation of DNBS colitis features, observed in our study, could be the consequence of the inhibition of early steps of inflammation. A reduced activation of NF-κB would lead to the infiltrating cells to decrease the amounts of inflammatory mediators and subsequently to preserve mucosal integrity. Once more, such an effect could be also positively related to the high concentration of C-phycocyanins present in the extract, which include C-phycocyanins + PEC (phycoerythrocyanins), and C-phycocyanins by themselves have been reported to be able to suppress activation of NF-κB in RAW 264.7 macrophages stimulated with LPS [41].

The DNBS model is also commonly associated with the T-helper (Th)1 response, and with an overproduction of proinflammatory cytokines, as IL-6 and IL 1β. Many of the IBD treatments act to regulate the levels of proinflammatory cytokines [42]. These immunoregulatory cytokines are involved in the initiation of the inflammatory response in colitis, amplifying the inflammatory reaction by triggering a cascade of immune cells, impairing intestinal permeability, and causing severe colonic infiltration [43] In addition, IL-1β contributes to the induction of the epithelial cell necrosis. Our results indicated that AphaMax^®^ treatment caused a dose-dependent reduction of both IL-6 and IL- 1β cytokine levels in colitis rats, thus ameliorating the deregulated immune response typical of experimental colitis. Once more, such an effect could be likely due to the high concentration of AFA C-phycocyanins.

Moreover, AphaMax^®^ significantly attenuated expression of the enzymes COX-2 and iNOS which were up-regulated in colitis rats. COX-2 is a key enzyme in the pathogenesis of IBD involved in the biosynthesis of prostaglandin [44], and target of many drugs used in the treatment of human IBD, including aminosalicylates [45,46]. This inhibition is not unexpected since C-phycocyanins are reported to be selective COX-2 inhibitors [31,47]. The expression of iNOS at sites of inflammation, acting in synergy with COX-2 to produce excessive inflammatory mediators has been reported. Their products may be detrimental to the colon integrity and contribute to the intestinal hypomotility and subsequent bacterial overgrowth [48], a typical features of the inflammatory reaction [49]. Accordingly, AphaMax^®^ treatment reduced nitrite levels in colitis rats. Considering that expression of both COX-2 and iNOS is regulated by NF-κB [50,51] the observed inhibition of NF-κB and its downstream effectors, COX-2 and iNOS, can be crucial for the protective role played by AphaMax^®^ treatment in DNBS colitis rats.

Lastly, in colorectal biopsies of human IBD [52,53] and in different experimental models of colitis, including the DNBS model, a mucosal production of reactive oxygen mediators was described, which would contribute to tissue damage during chronic intestinal inflammation [54].

The major effects of oxidative stress reported are local intestinal cell damage and activation of several signaling pathways, initiating inflammation [54].

In our preparations, AphaMax^®^ treatment was able to modulate the redox status by scavenging ROS and to reduce the severity of the colitis, effect that is likely ascribable to AFA-phycocyanins, which possess antioxidant activity 75 to 200 times greater than that other more common phycocyanins [14]. Phycocyanins were reported to be able to scavenge hydroxyl and alcoxyl radicals in an acetic acid-induced colitis model [36]. Thereafter, since free radical generation could contribute to the initial infiltration of neutrophils in the colonic mucosa, we can speculate that ability of our extract in preserving the colonic mucosa from oxidative insult could participate to decrease the neutrophil infiltration DNBS-induced. However, whether the antioxidative effect precedes the anti-inflammatory one or not is still uncertain and has yet to be explored.

We focused our attention on the AphaMax^®^ effects on the local production of ROS and oxidant insult in colonic tissue; however, since a plasmatic increase of advanced oxidation protein products formed during oxidative stress in IBD patients was also reported [55], further studies investigating possible AphaMax^®^ systemic effects are warranted.

## 5. Conclusions

In conclusion, taken together, data presented in this study demonstrate that AphaMax^®^ powder has pharmacologically promising positive activity on DNBS-mediated experimental colitis.

Although several mechanisms could be proposed to explain AphaMax^®^ beneficial effects, we highlight the reduction of inflammatory and oxidant mediators. However, further studies should be performed to resolve the exact molecular mechanisms. A limitation of this study is the lack of long-term observation following AphaMax^®^ treatment, since DNBS rats recovered within 10 days of the experiment, making it difficult to observe and compare the long-term effects of treatment on IBD. In addition, hapten-administration shares many features with human inflammatory bowel diseases, but the “acute” inflammatory response evoked should be different from the mechanism of chronic colitis in human IBD.

Moreover, the aim of the current study did not include the analysis of the effects of AphaMax^®^ treatment on the colon functions, we are aware that changes in colon motility and in intestinal permeability are described in DNBS-induced colitis in rats. Since it is well reported that antioxidant compounds are able to improve intestinal motility [56], additional studies could be performed to assess whether the mitigation of the inflammatory process by the AphaMax^®^ treatment, could help the colonic mucosa and the smooth muscle to recover their functionality. We hope that results of this study can prompt further investigations on AphaMax^®^ as a natural product in the management of intestinal inflammatory diseases.

## Figures and Tables

**Table 1:** Primers used for qRT-PCR.

Gene Description	Forward Primer	Reverse Primer	Ref.
β-actin	5′-CTAAGGCCAACCGTGAAAAG-3′	5′-GCCTGGATGGCTACGTACA-3′	[26]
COX 2	5′-TGCGATGCTCTTCCGAGCTGTGCT-3′	5′-TCAGGAAGTTCCTTATTTCCTTTC-3′	[27]
iNOS	5′-AGAAGGGGACGAACTCAGC-3′	5′-TCGAACATCGAACGTCTCAC-3′	[28]
NFκ−B p65	5′-GTCATCAGGAAGAGGTTTGGCT-3′	5′-TGATAAGCTTAGCCCTTGCAGC-3′	[29]

**Table 2:** Effects of Aphamax^®^ on body weight change, stool consistency, rectal bleeding 2- and 7- days after colitis-induction.

Groups	% Body Weight Change 2 Days after Colitis Induction	% Body Weight Change 7 Days After Colitis Induction	Stool Consistency 2 Days after Colitis Induction	Stool Consistency 7 Days after Colitis Induction	Rectal Bleeding 2 Days after Colitis Induction	Rectal Bleeding 7 Days after Colitis Induction
**Sham**	102.1 ± 0.5	109 ± 0.8	0	0	0	0
**Sham +** **100 mg/kg Aphamax^®^**	103.2 ± 1.2	107 ±1.2	0	0	0	0
**DNBS**	94.2 ± 0.8 *	95.1 ± 1.5 *	4.0 ± 0.0 *	3.8 ± 0.2 *	4.0 ± 0.0 *	3.8 ± 0.2 *
**DNBS+** **20 mg/kg Aphamax^®^**	95.4 ± 1.3 *	99.2 ± 0.9 *	3.2 ± 0.2 *	2.8 ± 0.2 *	3.2 ± 0.4 *	2.0 ± 0.3 * ^§^
**DNBS+** **50 mg/kg Aphamax^®^**	96.7 ± 0.9 *	102.2 ± 0.5 ^§^	2.8 ± 0.2 *	2.0 ± 0.4 * ^§^	2.2 ± 0.4 * ^§^	1.2 ± 0.2 ^§^
**DNBS+** **100 mg/kg Aphamax^®^-**	97.8 ± 1.1 *	104.4 ± 1.5 ^§^	2.7 ± 0.4 * ^§^	1.0 ± 0.3 ^§^	2.0 ± 0.6 * ^§^	0.4 ± 0.2 ^§^

Percent of body weight change compared to the original body weight (taken as 100%), consistency of the stool rectal bleeding scored following Cooper et al. [22] method in sham rats or in 20, 50, and 100 mg/kg Aphamax^®^- treated rats with or without colitis induction. Data are means ± S.E.M. *n* = 5 animals/ each group. * *p* < 0.05 *versus* Sham group; ^§^ *p* < 0.05 *versus* 2,4 dinitrobenzensulfonic acid (DNBS) group.
